# Orodispersible Dosage Forms with Rhinacanthin-Rich Extract as a Convenient Formulation Dedicated to Pediatric Patients

**DOI:** 10.3390/ph17080994

**Published:** 2024-07-27

**Authors:** Thongtham Suksawat, Witold Brniak, Ewelina Łyszczarz, Małgorzata Wesoły, Patrycja Ciosek-Skibińska, Aleksander Mendyk

**Affiliations:** 1 Faculty of Pharmacy, Mahidol University, Bangkok 10400, Thailand; 2 Department of Pharmaceutical Technology and Biopharmaceutics, Faculty of Pharmacy, Jagiellonian University Medical College, Medyczna 9, 30-688 Krakow, Poland; 3 Chair of Medical Biotechnology, Warsaw University of Technology, Noakowskiego 3, 00-664 Warsaw, Poland

**Keywords:** orodispersible tablets, orally disintegrating tablets, orodispersible films, rhinacanthin, taste masking, electronic tongue, pediatric pharmacotherapy, herbal medicines

## Abstract

Rhinacanthins, derived from *Rhinacanthus nasutus*, widely used in traditional medicine, exhibit antifungal, anticancer, antiviral, antibacterial, and antiplatelet aggregation effects. Recently, their anti-diabetic activity was confirmed, which makes them an interesting natural alternative in the therapy of the early stage of diabetes mellitus. The aim of this study was to demonstrate the possibility of formulating orodispersible tablets (ODTs) and orodispersible films (ODFs) containing rhinacanthin-rich extract (RRE). Tablets with 50 mg or 100 mg of RRE were produced by direct compression. ODFs were manufactured by casting of Lycoat RS 720 or polyvinyl alcohol solution with RRE and additional excipients. The mechanical properties and disintegration times of the prepared formulations were studied. The effectiveness of taste masking was analyzed with an electronic tongue system. Six months simplified stability studies were performed in conditions complying to ICH guidelines. Appropriate friability of ODTs was achieved, despite low tensile strength (0.45–0.62 MPa). All prepared ODFs successfully met the acceptance criteria regarding Young’s modulus, tensile strength, and elongation at break. The observed variations in their mechanical properties were dependent on the type and quantity of polymers and plasticizers used. Disintegration time of ODTs ranged from 38.7 s to 54.2 s, while for ODFs from 24.2 to 40 s in the pharmacopoeial apparatus. Analyses made with the electronic tongue showed the significant taste-masking effect in both formulations. The addition of sucralose as a sweetener and menthol with mint flavor as a taste-masking agent was sufficient to mask an RRE’s taste in the case of ODTs and ODFs. Stability studies of ODTs packed in the PVC/Alu blisters showed a decrease in the RRE content below 90% after 6 months. However, ODFs with PVA were physicochemically stable for 6 months while being stored in Alu/Alu sachets. Our study proved for the first time the possibility of the formulation of orodispersible dosage forms with RRE, characterized by good mechanical properties, disintegration time, and appropriate taste masking.

## 1. Introduction

Despite many challenges encountered in the utilization of solid oral dosage forms in pharmacotherapy, this mode of drug delivery is still the most popular way of administration. Swallowing difficulties, particularly prevalent among pediatric populations, as well as individuals with disabilities or those confined to bed, contribute to the complexity of oral drug administration. Efforts to develop satisfactory formulations for children, despite governmental incentives, remains notably challenging. These challenges are exacerbated by factors such as the pervasive poor solubility and absorption of active pharmaceutical ingredients, resulting in their insufficient bioavailability. Furthermore, specific pediatric formulations exhibit significant changes in required dose strength, and achieving swallowing appropriateness proves particularly burdensome for children under the age of 10. The incorporation of unpalatable tastes associated with certain compounds or excipients further complicates formulation efforts [1,2].

At the same time, oral administration remains the dominant method of drug delivery, underpinned by its non-invasive, simplistic, and convenient application. Oral solid dosage forms offer inherent advantages, including dose accuracy, relatively high stability, and the potential to modulate drug release profiles to facilitate delayed or sustained therapeutic effects [3,4,5,6].

Addressing the challenges affiliated with swallowing solid dosage forms entails the exploration of orodispersible formulations, such as orodispersible tablets (also called orally disintegrating tablets—ODTs) and orally disintegrating films (ODFs). ODTs are uncoated tablets designed to be placed in the oral cavity where they quickly disperse before being swallowed. They may feature immediate release dosage form as well as be modified release advanced formulations containing microparticles, pellets, or granules coated with functional excipients. Although originally they were manufactured with freeze-drying processes, nowadays they are mostly produced by the direct compression of powders [7]. On the other hand, novel advanced technological methods such as 3D printing are also utilized in the production of ODTs [8,9,10,11].

ODFs are post-stamp size thin polymer sheets, made of water-soluble polymers such as poly(vinyl alcohol) (PVA), cellulose derivates (hydroxypropyl methylcellulose, HPMC), and starch derivates (maltodextrins, hydroxypropyl starch) [12]. Various methods have been applied to their preparation, such as inject or flexographic printing; 3D printing; electrospinning; hot melt extrusion; and solvent casting, which is the most commonly used technique, both on a lab-scale as well as an industrial continuous production [2,13,14]. In this method, an active pharmaceutical ingredient (API) is dissolved or dispersed in a polymeric solution with other excipients, e.g., plasticizers, sweeteners, and flavors. After deaeration, casting solution/suspension is cast with defined thickness, dry at room or high temperature, and cut into strips.

Nevertheless, not only the dosage form but also an appropriate taste affects the overall acceptability of medicinal products. Many research studies are focused on effective taste masking of poor taste of drug substances in orodispersible dosage forms, e.g., complexation with cyclodextrins, addition of sweeteners, or drug microencapsulation [15,16,17]. Among the taste-masking agents, the most commonly used in orodispersible formulations are sucralose, sorbitol, xylitol, acesulfame K, menthol, mint, and licorice [18,19,20]. Their effectiveness may be evaluated utilizing electronic tongue [15,21].

**Figure 1:**
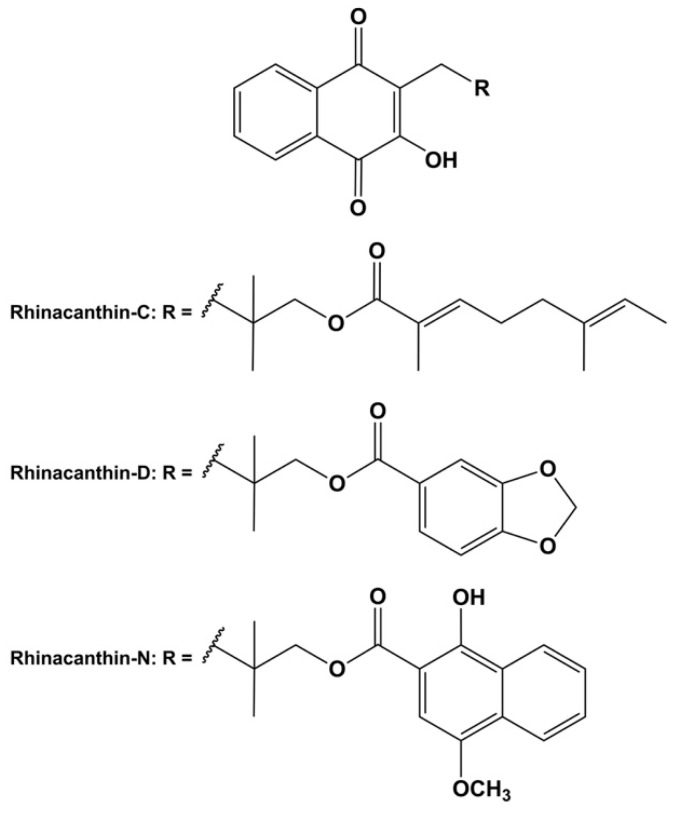
Chemical structures of rhinacanthin-C, -D, and -N.

Rhinacanthins, specifically rhinacanthin-C, rhinacanthin-D, and rhinacanthin-N (Figure 1), represent biologically active compounds derived from *Rhinacanthus nasutus* [22,23,24,25]. Widely employed in herbal medicine, these substances exhibit diverse pharmacological properties, encompassing antifungal, anticancer, antiviral, antibacterial, and antiplatelet aggregation effects [25,26]. Recent investigations have unveiled their potential as anti-diabetic agents, showcasing promising efficacy in both in vitro and in vivo studies [24]. Consequently, rhinacanthins are recognized as potential naturally occurring chemicals for anti-diabetic interventions.

In an innovative approach, a green methodology has been successfully employed for the preparation of rhinacanthin-rich extract (RRE) as an alternative to pure rhinacanthins for anti-diabetic assessments [25]. However, RRE has demonstrated significantly poor water solubility, posing challenges to its bioavailability. To surmount these limitations associated with pure solubility of rhinacanthins and β-cyclodextrin, a ternary inclusion complex has been devised, involving the formation of an intricate molecular structure between RRE, β-cyclodextrin, and PVP K30 [25]. This strategic development aims to enhance the solubility and possibly bioavailability of RRE, thereby advancing its potential as a viable anti-diabetic therapeutic agent. Considering the challenges associated with swallowing conventional dosage forms, such as tablets or capsules, influenced by diseases and the active pharmaceutical ingredients (API) employed in their treatment, a potential risk arises for non-compliance or discontinuation of pharmacotherapy. Consequently, it is suggested to explore the development of more palatable dosage forms incorporating rhinacanthin extract, such as ODTs or ODFs. Natural compounds have previously been incorporated to orodispersible dosage forms [27,28,29,30]; however, there is still a significant gap to be filled in the development of such forms. Their availability, especially in western countries, is very low, even despite of the growing demands. 

The objective of the current investigation was to formulate and assess two orodispersible dosage forms, specifically ODTs and ODFs, incorporating a ternary complex of RRE. We assessed the effect of sweetener and flavorants, i.e., sucralose, mint flavor, and menthol, on the taste of the final drug forms using an electronic tongue. In the case of ODTs, tabletability, mechanical properties, disintegration time, and stability were analyzed. In the case of ODFs, the influence of film-forming polymers and plasticizer concentration on mechanical properties, wettability, and disintegration time was evaluated.

## 2. Results

### 2.1. Preparation of Ternary Complex of Rhinacanthin-Rich Extract (RRE)

Ternary complex of RRE was prepared according to the previously described procedure [31]. The total rhinacanthin contents in RRE and ternary complexes of RRE were 86.08 ± 2.55% and 23.18 ± 0.19% *w*/*w*, respectively.

### 2.2. Preparation of Tablets

Tableting of plant origin materials can pose serious technological problems, because of the poor compressibility characteristics and wide variations in the material properties. Therefore, on the very first stage of the ODT development process, we prepared several dozen series of so called preformulations (preliminary formulations) to evaluate the possibility of compressing RRE to the form of tablets with acceptable properties. At this stage, only mechanical properties and disintegration time were measured, as well as the overall ability of the prepared powder mixes for the tableting (Table A1). Prepared tablets had mass from 260 mg to 450 mg and diameter from 9 mm to 12 mm. Two different doses of RRE were used, namely, 50 mg or 100 mg. Four different co-processed excipients dedicated for the direct compression of ODTs were used as a ready-to-use platforms to prepare tablet masses (F-Melt type C, Ludiflash, Pharmaburst and Ultraburst). None of them was appropriate to achieve tablets with satisfactory properties, and in each case, additional excipients were needed. We evaluated two different grades of microcrystalline cellulose, spray-dried lactose, and D-mannitol as fillers improving compressibility of the powders. Crospovidone, croscarmellose sodium, or sodium starch glycolate were added as superdisintegrants to speed up the disintegration process. Calcium silicate or magnesium aluminometasilicate were used to additionally improve compressibility, flowability, and tabletability of the powders. Due to the poor flowability of RRE, silicone dioxide was added to each formulation in the amount of 1–2%. Sodium stearyl fumarate, which is recommended as a lubricant in ODTs, was used in the amount of 1–4%. Additionally, several different flavors, sweeteners, and taste-masking agents were combined to mask the unpleasant taste of RRE.

The differences among parameters of preliminary tablets were huge (Table A2). Their hardness ranged from as low as 13.1 N to as high as 129.8 N. Disintegration time of 9 series out of 32 were longer than 3 min, which means that they could not even be considered as an ODT. This was the main reason for employing additional excipients apart from the co-processed platforms. The shortest disintegration times were noticed in the formulations with Ultraburst and Pharmaburst. The latter was finally selected for the formulation, because Ultraburst has a little effervescent effect which was undesirable in our formulations. Disintegration time of tablets with Pharmaburst ranged from 39.6 s to 204.6 s and was strongly dependent on the addition of other excipients. There was no direct correlation between hardness and disintegration time.

Based on these preliminary results, two series with different doses of RRE were selected for the further studies, i.e., F20 and F32 (Table A1). Bigger batches of these formulations were prepared using rotary tablet press. For the easier differentiation, they were named T12_100 mg and T10_50 mg, correspondingly (see Section 4.3). During the compression process, die volume was adjusted by the regulation of lower punch position in order to maintain constant tablet mass. The hardness of tablets was monitored throughout the process to maintain it in the range of 30–40 N. The upper punch position was adjusted if needed. No capping, punch sticking, or other compression problems were noticed during the process.

### 2.3. Properties of Tablets

Evaluation of tablets’ properties was based on their mechanical parameters, disintegration time measurements, and analysis of their uniformity (Table 1).

#### 2.3.1. Mechanical Characteristic

Hardness of both formulations was very similar, despite the difference in size and diameter. Hardness of 10 mm tablets was 32.1 N and tablets of 12 mm diameter was 30.1 N (Table 1). In order to compare them, we also calculated tensile strength according to the USP requirements [32]. This parameter includes not only hardness (resistance to crushing) but also thickness and diameter of the tablets. Therefore, if there are differences in the sizes of compared tablets, it is more relevant than hardness. Tensile strength was 0.62 MPa in the case of smaller tablets and 0.45 MPa in the case of bigger tablets. Both of them are relatively low, which resulted from the poor compressibility of RRE. Increasing of compression force during tableting process did not increase hardness of tablets, but rather caused capping effect and worsening of their mechanical properties. Therefore, we compressed them with relatively low force to avoid this phenomenon. 

Despite the low hardness, the friability of both formulations complied with the Eur. Pharm. and USP limit of 1% [33,34]. In the case of 10 mm tablets, it was 0.75%, and in the case of 12 mm tablets, 0.53%.

#### 2.3.2. Disintegration of ODTs

Disintegration time was measured with two different methods: compendial and alternative, more biorelevant, in the BJKSN apparatus developed in our department [35] and previously used in multiple research studies [17]. In the pharmacopoeial test, smaller tablets disintegrated within 38.7 s, while bigger in 54.2 s, which fully complied with the European Pharmacopoeia limit of 3 min. However, disintegration times measured in the BJKSN apparatus were much higher. In the case of smaller tablets, it was 137.1 s, and in the case of bigger tablets, over 3 min. These differences resulted from different volumes of media used in these two tests. In the pharmacopoeial method, approximately 800 mL of water is used, but in the BJKSN apparatus, only 0.8 mL of water moistens the tablet. If the main mechanism of tablet disintegration involves dissolving of excipients or API, such a small volume may be insufficient for quick disintegration.

#### 2.3.3. Uniformity of Tablets

The uniformity of the prepared tablets was evaluated based on three different approaches: mass deviation, content deviation, and uniformity of dosage units. All of them indicated very narrow deviations from mass or content for both sizes of tablets. The maximum allowed deviation of tablet mass according to the monograph 2.9.5 of the Eur. Pharm. 11.0 is ±5% in the case of tablets with an average mass ≥ 250 mg [36]. Deviation of the mass of a single 10 mm tablet from the average value was in the range of 0.25–2.78%, while in the case of 12 mm tablets, it was 0.06–3.20%, which fully complied with pharmacopoeial requirement.

Average content of RRE in 10 mm tablets was 49.85 mg, and in 12 mm tablets, 99.67 mg. Standard deviations of content were 1.57 mg and 0.78 mg, respectively, and a difference from the average content reached from 0.06% to 2.64% in single tablets. The maximum deviation for the content, according to the monograph 2.9.6 of the Eur. Pharm. [37] is ±15%, which indicates that prepared tablets were very uniform. 

We also evaluated the uniformity of tablets according to the monograph 2.9.40 [38]. In the case of prepared tablets, this evaluation should be based on the content uniformity; however, if the standard deviation for content is below 2%, it may be alternatively based on the mass variation test. Therefore, we performed both analyses. Acceptance values calculated based on the content uniformity were 1.88 and 1.87 for 10 mm and 12 mm tablets, respectively. Slightly higher values were obtained when mass variations were considered, i.e., 2.10 and 4.35; however, all of them were still far below the pharmacopoeial limit, which is not more than 15.

In sum, both ODT formulations fulfilled all requirements of the European Pharmacopoeia 11.0 [36,37,38] for such a dosage form. 

### 2.4. Preparation of Films

In preliminary studies, we pre-selected the amount and type of plasticizer, i.e., glycerol and sorbitol, as well as the concentration of the film-forming polymer. ODF formulations incorporating glycerol at concentrations ranging from 3 to 10% exhibited fragility and brittleness. In contrast, films containing sorbitol demonstrated increased flexibility and durability. However, formulations exceeding 20% sorbitol content displayed excessive ductility and stickiness. Consequently, for subsequent investigations, only formulations comprising 25% Lycoat or PVA, 10–20% sorbitol, and 10% of the ternary complex of RRE were selected for in-depth analysis to determine the optimal formulations. The film parameters are presented in Table 2, revealing that films containing the ternary complex of RRE exhibited a thickness ranging from 0.207 mm to 0.231 mm and an average weight of 0.148–0.184 g.

### 2.5. Mechanical Parameters of Films

**Figure 2:**
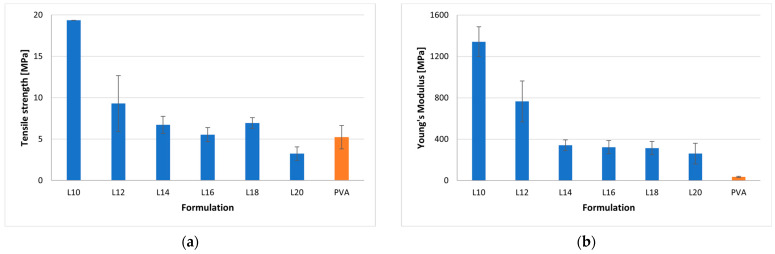
Mechanical properties of the ODFs based on the Lycoat RS 720—blue color, and PVA—orange color, where (**a**) tensile strength is contained in the first panel, and (**b**) Young’s modulus is contained in the second panel.

Table 2 and Figure 2 comprehensively outline the mechanical characteristics of ODFs, encompassing the percentage of elongation, tensile strength, and Young’s modulus. Upon comparing various plasticizer types, it was observed that films incorporating sorbitol exhibited superior strength, flexibility, extensibility, and durability compared to films employing glycerol as a plasticizer. Films plasticized with glycerol, conversely, displayed characteristics of fragility, brittleness, and hardness. Specifically, Formulation L10, plasticized with 10% sorbitol, demonstrated the highest Young’s modulus value (1343.00 MPa) and tensile strength (19.36 MPa), along with the lowest percentages of elongation (1.97%), when juxtaposed with other film formulations. Moreover, higher concentrations of sorbitol in formulations L12, L14, L16, L18, and L20 (ranging from 12% to 20%) resulted in a decrease in tensile strength and Young’s modulus, coupled with a decrease in the value of percentage of elongation. Formulation L20, incorporating 20% sorbitol, exhibited the highest percentage of elongation (39.15%) and the lowest Young’s modulus (260.7 MPa). These films demonstrated a moderate tensile strength of 3.25 MPa. Despite these favorable mechanical properties, it was noted that the L20 formulation was excessively ductile and sticky, resulting in challenges during the peeling process from the foil. Consequently, only Formulation L16, which also displayed commendable mechanical characteristics, namely, moderate tensile strength and low Young’s modulus, was chosen for subsequent investigations. It is noteworthy that the PVA-based formulation retained commendable mechanical properties, featuring a percentage of elongation (94.23%), Young’s modulus (35.5 MPa), and tensile strength (5.23 MPa).

### 2.6. Contact Angle of Films

The wettability of ODFs is influenced by the inherent characteristics of the film-forming polymer, the type of plasticizer employed, and the solubility of the API. In formulations utilizing Lycoat as the base, an escalation in the quantity of the plasticizer (sorbitol) within the range of 10–20% resulted in a decrease in the contact angle, indicative of heightened hydrophilicity on the film surface (Table 3). Notably, Formulation L20 exhibited the lowest contact angle at 62.0°, suggesting a highly hydrophilic surface. For other systems, contact angle values exceeded 65°–70°, signifying a more hydrophobic nature in the chemical composition of the films.

However, it is worth mentioning that after the 10th measurement point, formulations L18 and L20 displayed the lowest contact angles of 48.8° and 49.2°, respectively. Although these differences are statistically significant (*p* < 0.05), there is no linear relationship between the contact angle value and the amount of plasticizer, but rather nuanced variations between formulations. In contrast to Lycoat-based formulations, those based on PVA exhibited significantly lower contact angles (*p* < 0.05) at both the 1st and 10th measurement points (57.0° and 47.3°, respectively). 

### 2.7. Disintegration of Films

**Figure 3:**
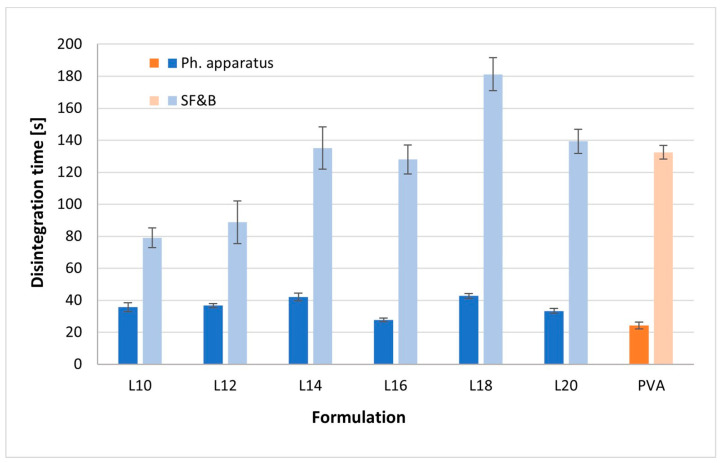
The impact of the sorbitol content on the disintegration time of the ODFs based on the Lycoat RS 720—blue color and PVA—orange color.

The mean disintegration times, obtained through both a pharmacopoeial apparatus and a slide frame and ball method (SF&B), are delineated in Figure 3. It is noteworthy that the disintegration time measured using the pharmacopoeial method was significantly shorter than that determined using the slide frame and ball method. However, the latter method exhibited higher sensitivity to variations in film thickness. Across all film types, the mean disintegration time remained below 60 s for the pharmacopoeial method and under 200 s for the slide frame and ball method.

In the case of the Lycoat-based formulation, the slide frame and ball method revealed an increasing trend in disintegration time with the augmentation of sorbitol content between 10% and 18% (79.0–181.2 s). However, at 20% sorbitol, the disintegration time significantly decreased (139.3 s). In the pharmacopoeial test, the lowest disintegration time was observed in Formulation L16 (27.7 s). Consequently, both methods were employed to validate the optimal plasticizer concentration in the formulation, indicating the range of 16% to 18% sorbitol. 

The PVA-based formulation maintained a favorable disintegration profile in both methods, with a pharmacopoeial disintegration time of 24.2 s and a slide frame and ball method disintegration time of 132.4 s.

### 2.8. Electronic Tongue Studies—Evaluation of Taste-Masking Efficiency in RRE ODFs and ODTs upon the Addition of the Sweetener and Flavorants

Principal component analysis (PCA) is a linear dimensionality reduction technique applied to explore the sensor array data, which are multidimensional. The data are linearly transformed onto a new coordinate system. The obtained, new directions are called principal components (PCs), and their characteristic feature is that they capture the largest available variation of the original data in the following PCs. As a result of transforming the original matrix of the sensor array signals onto this new PCA subspace, the first principal component PC1 will have the largest possible variance, and all consequent principal components (PC2, PC3, and so on) will have the largest variance given the constraint that these components are uncorrelated (orthogonal) to the other principal components. 

The ISEs applied in the sensor array exhibited high sensitivity towards RRE (compare description in Section 4.12.1), and therefore it was expected that information contained in the first PCs (especially in PC1-PC2 space) carries information related to the amount of the released API and enables the observation of the eventual taste-masking effect.

**Figure 4:**
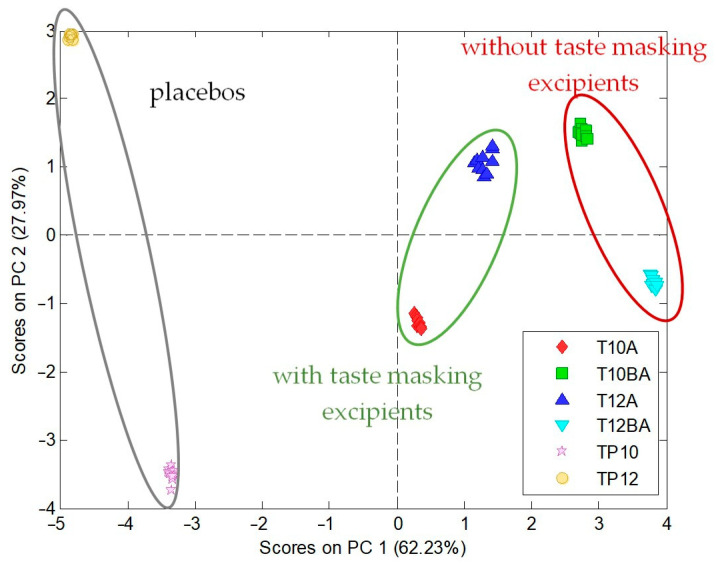
P2. PCA score plot of electronic tongue results for RRE ODTs: T10A, T12A–T10, and T12 ODTs with taste-masking excipient addition; T10BA, T12BA–T10, and T12 ODTs without taste masking excipients addition; TP10, TP12–T10, and T12 ODT placebos.

The prepared sensor array of e-tongue was applied to check taste-masking efficiency in the studied formulations, both ODTs and ODFs. Such a test relies on the comparison between various formulations. For tablets, two sizes (T10 and T12) were considered: without the addition of taste-masking excipients, i.e., sucralose, mint flavor, and menthol (T10BA, T12BA), and with them (T10A, T12A). They were compared with respective placebos (TP10, TP12). Thus, six types of samples were tested according to the procedure described in the experimental section. Electronic tongue results in the form of PCA score plot are presented in Figure 4. 

All samples formed easily separable, distinct clusters. ODTs containing RRE are easily discernable from placebos (without RRE), and they even have opposite values of PC1. This can be regarded as having different chemical images, suggesting the appearance of a bitter taste. Chemical images of T10BA, T12BA, T10A, and T12A recorded by electronic tongue are easily separable on the PCA plot, which can be related to taste-masking effect of varying degrees. Moreover, the addition of the sweetener and flavorants in ODTs of both sizes caused the reduction of the bitterness because chemical images of ODTs with the taste-masking excipient addition (T10A and T12A) are closer to chemical images of placebos (TP10 and TP12) than chemical images of ODTs without the addition of taste-masking excipients. 

**Figure 5:**
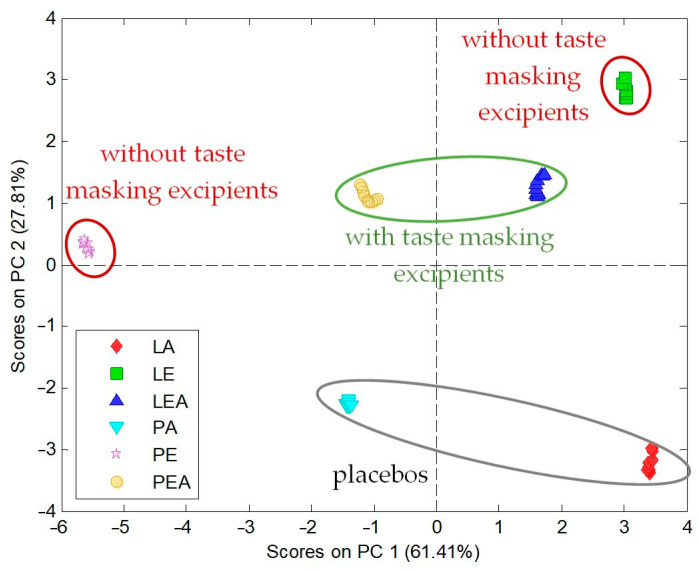
PCA score plot of electronic tongue results for RRE ODFs: LEA and PEA–Lycoat and PVA-based films with sweetener addition; LE and PE–Lycoat and PVA-based films without sweetener addition; LA and PA-Lycoat and PVA-based films without RRE (placebos).

In the case of ODFs, six types of samples were also tested by the electronic tongue, which were based on Lycoat (L) and PVA (P) polymers: RRE ODFs prepared with the addition of taste-masking excipients (LEA and PEA) and without them (LE and PE), and for comparison, ODFs not containing RRE (placebos, LA, and PA). (Dis)similarity of the tested samples was evaluated by means of PCA, and the resulting PCA score plot is presented in Figure 5.

Again, as in the case of ODTs, easily separable, distinct clusters were observed for all samples. Both placebos were characterized by a negative value of PC2, in contrast to formulations with RRE (such a distinction was observed also in the case of ODTs but regarding PC1). Along PC1, clear distinction between chemical images of formulations based on different polymers can be observed—all Lycoat-based ODFs were characterized by a positive value of PC1, while PVA-based ODFs by a negative value of PC1. For both polymers, films with and without taste-masking excipients were easily discernable from placebos; moreover, ODFs with the addition of taste-masking excipients were closer to placebos than respective formulations not containing taste-masking agents. Therefore, it can be concluded that electronic tongue results suggest that the addition of the sucralose, mint flavor, and menthol probably provides a taste-masking effect, both in RRE ODTs and RRE ODFs. 

### 2.9. Stability Studies

Simplified stability studies were performed for the period of 6 months for both kinds of formulations, i.e., ODTs and ODFs. They were packed in two different types of packaging to additionally check the effect of the container on the stability of dosage forms containing RRE. In the case of ODTs, standard PVC/Alu blisters with low barrier properties were used, while the ODFs were enclosed in the sealed aluminum blisters impermeable for the humidity and oxygen. 

#### 2.9.1. Stability of ODTs

**Figure 6:**
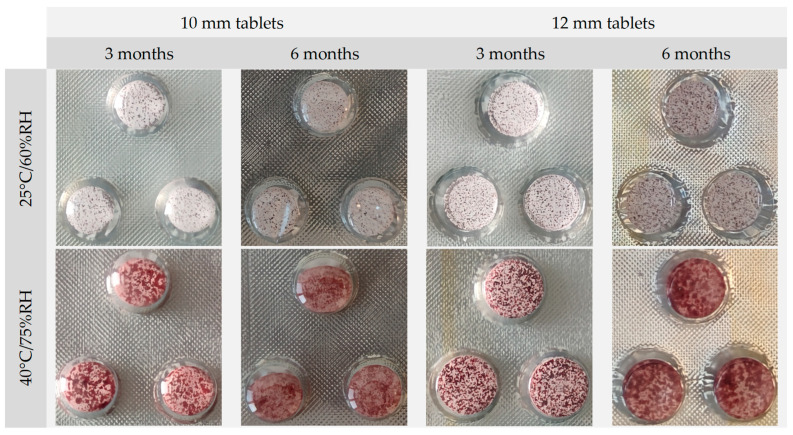
Appearance of the ODTs after storage for 3 and 6 months during the stability studies.

The appearance of the RRE is not uniform. It is mostly red powder but with a considerable amount of pale white particles. Therefore, the surface of the tablets was also not uniform, and there were clearly visible multiple red or burgundy spots scattered across the entire surface of tablets. These spots did not form agglomerates but were rather uniformly distributed over the entire surface. The appearance of the tablets of both sizes did not change after 3 months of storage in the 25 °C/60%RH, but after 6 months, some spots became slightly bigger (Figure 6). It was more visible in the case of 12 mm tablets, i.e., containing a higher dose of RRE.

The changes of appearance of both kinds of ODTs stored in a higher temperature and humidity were noticed after just 1 month. These changes were even more visible after 3 and 6 months. The small red spots on the surface were spread in the form of red stains covering almost the whole surface. The structure of the tablet also changed. Their volume increased, and in some cases, tablets filled whole blister cavities. These changes could result from the high humidity in the chambers and the fact that PVC/Alu blisters are rather permeable and did not form enough barrier for the water vapor. 

**Figure 7:**
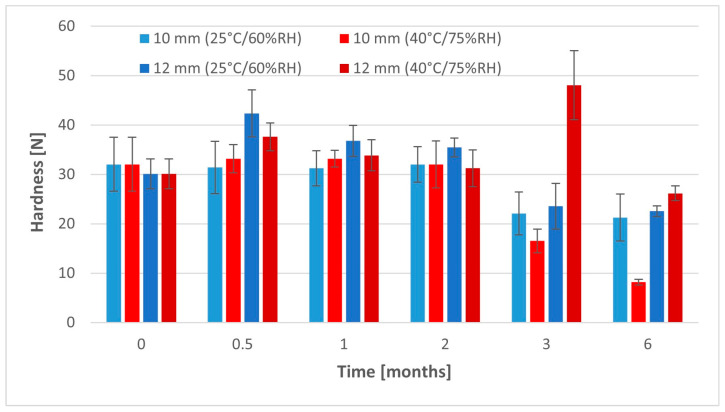
Hardness of tablets during stability studies.

The mechanical properties of 10 mm ODTs did not change significantly for the first 2 months, despite the conditions of storage (Figure 7, Table A3). However, the hardness of the 12 mm ODTs after this period was significantly higher (*p* < 0.05). The values of hardness for all tablets ranged from 30.08 N to 42.35 N in this period (Table A4). After a longer storage time, in the case of tablets stored in the lower temperature, we noticed a gradual worsening of the mechanical parameters. The hardness decreased after 3 months to 22.07–23.54 N and after 6 months to 21.26–22.56 N, which was significantly lower than directly after preparation. Despite that change, hardness was still sufficient for smaller tablets to withstand the friability test. The mass loss was 0.75%, which was exactly the same value as directly after compression. On the other hand, the bigger tablets performed much worse in the friability test after 6 months of storage. A couple of them had the tendency towards capping, which finally led to a failure of this test (mass loss > 1%).

Hardness of the 10 mm tablets stored in the higher temperature and humidity decreased after 3 and 6 months, even more than after storage in milder conditions. Its value dropped to 16.51 N after 3 months and to 8.18 N after 6 months (Table A3). These values were significantly lower than for tablets stored at a temperature of 25 °C. In the case of bigger tablets, the hardness increased after 3 months to 48.07 N, but after 6 months, we noticed a significantly lower value, i.e., 26.16 N (Table A4). Due to the absorption of a great amount of humid, tablets became softer, but at the same time more flexible and less fragile. This resulted in the lower value of friability after 6 months (0.57%), even though the hardness became lower.

**Figure 8:**
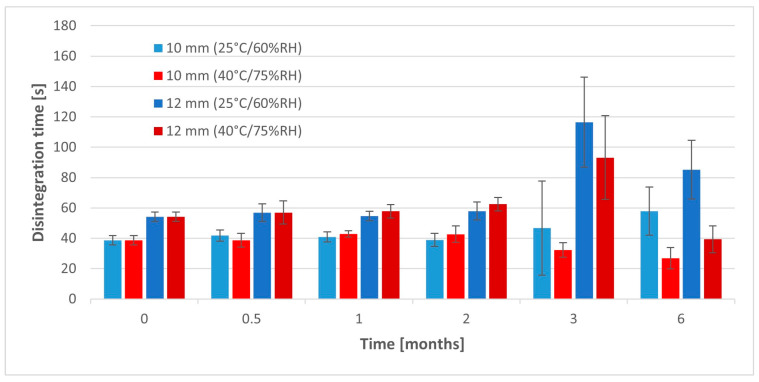
Disintegration of tablets in stability studies (pharmacopoeial method).

Disintegration time of 10 mm tablets stored in the lower temperature measured with a pharmacopoeial apparatus significantly increased over 6 months (Figure 8, Table A3). However, much higher standard deviations were noticed in the case of tablets analyzed after 3 and 6 months, which was a result of the sticking of tablets to the disks of the apparatus and increasing disintegration time in some samples. Disintegration time of 10 mm tablets stored at a higher temperature significantly decreased after 3 and 6 months from 38.7 s to 32.3 and 26.8 s respectively, which could be the result of the worsening of their mechanical resistance. 

The opposite relationships were found in the 12 mm tablets stored in the lower temperature. Their disintegration time significantly increased after 3 and 6 months (*p* < 0.05) (Table A4). We also noticed a similar increase in deviations of single samples due to the sticking of a tablet mass to the disks during the test. It caused problems with determination of the test and relatively higher values than in the previous tests. When these bigger tablets were stored in the higher temperature for 3 and 6 months, they disintegrated, forming much larger particles than after their preparations. There were multiple agglomerates of several millimeters floating in the disintegration medium, which was not previously presented. It suggests that superdisintegrants’ properties were significantly weakened due to the moisture absorption by the tablet mass. 

**Figure 9:**
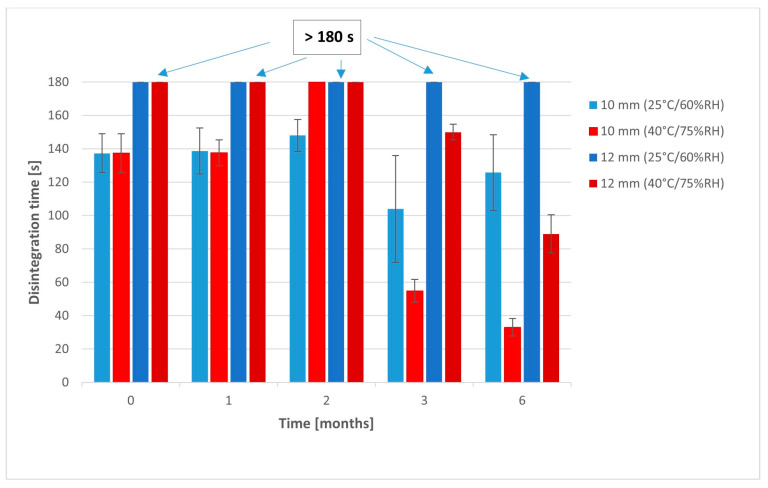
Disintegration of tablets in stability studies (BJKSN apparatus [35]).

Slightly different relationships were found in the disintegration test performed with the BJKSN apparatus designed as a biorelevant test imitating conditions in the oral cavity (Figure 9, Table A3 and Table A4). Due to the much lower volume of the medium used in this device than in the pharmacopoeial test, disintegration times measured with this apparatus are usually much longer than in the compendial method. On the other hand, this test is more discriminatory, due to the milder conditions and more mechanisms involved in the tablet disintegration. 

Disintegration times of 10 mm tablets stored in both conditions decreased after 3 and 6 months, which could be the outcome of the worsening of their mechanical parameters; however, these changes were not significant due to the high deviations of the results. ODTs with a 12 mm diameter had a much higher mass and their disintegration time was much longer, exceeding even 3 min. After 3 and 6 months of storage in the higher temperature, mechanical parameters of these tablets were much worse, and their disintegration time dropped below 3 min. 

**Figure 10:**
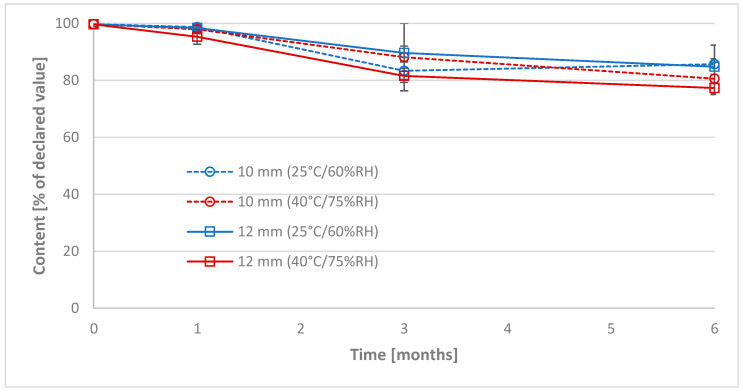
Content of RRE in prepared tablets during stability studies.

Content of the RRE in the prepared tablets was measured in 10 tablets of each formulation after 1 month, 3 months, and 6 months. We observed a steady decrease in the RRE over time (Figure 10, Table A3 and Table A4). This difference became significant (*p* < 0.05) after 3 months of storage. The degradation process was slightly faster when tablets were stored at higher temperatures. In the tablets stored at 25 °C/60%RH, the amount of API dropped to 85–86% after 6 months, while in tablets stored at 40 °C/75%RH, it decreased to 77–81% of the content at the beginning of stability tests.

#### 2.9.2. Stability of ODFs

**Figure A1:**
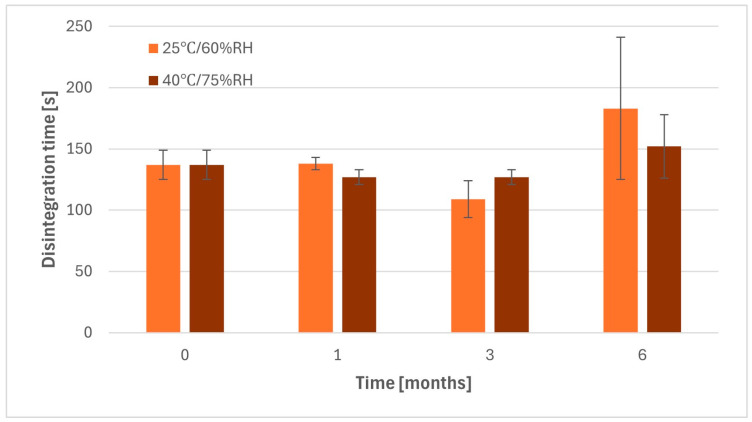
Disintegration time of PVA-based ODFs in stability test.

**Figure A2:**
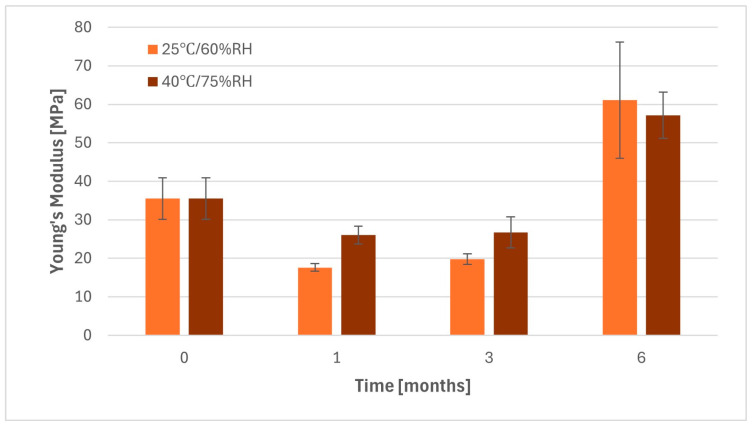
Young’s modulus of PVA-based ODFs in stability test.

**Figure A3:**
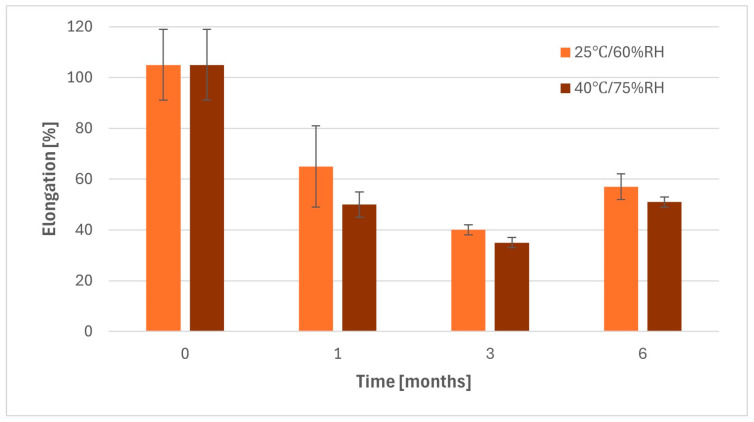
Elongation of PVA-based ODFs in stability test.

**Figure A4:**
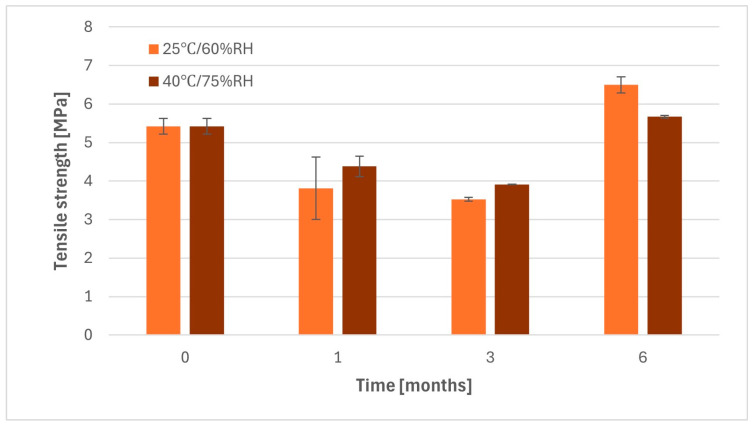
Tensile strength of PVA-based ODFs in stability test.

The stability assessment was conducted under conditions of 25 °C/60% RH and 40 °C/75% RH for a duration of 6 months (Table 4, Figure A1, Figure A2, Figure A3 and Figure A4). The evaluation encompassed the analysis of the physical, mechanical, and chemical attributes of the films at intervals of 0, 1, 3, and 6 months. The Lycoat-based films were very sticky, even after 1 month of storage, which made it impossible to separate them from each other and perform analyses. Therefore, the tests were carried out only for PVA-based films.

Throughout the stability study, the physical properties of the films remained consistent, with a mass ranging from 0.145 to 0.149 g, except at the 6 month mark under 40 °C/75% RH, where an increase in film weight was observed, reaching 0.161 g. Similarly, films subjected to 40 °C/75% RH at the 6 month interval exhibited distinct characteristics compared to films in other conditions, displaying a significantly higher thickness of 0.244 mm.

The disintegration time of films varied significantly in samples analyzed during the 6 month studies and under all stability study conditions, ranging from 109 to 183 s, but there was no steady trend of its changes in both conditions (Figure A1). Mechanical properties, including Young’s modulus (17.6–61.1 MPa), %Elongation (35–105%), and tensile strength (3.52–6.50 MPa), also exhibited some variations across all stability study conditions (Figure A2, Figure A3 and Figure A4). Despite the fact that some of them changed significantly, all values exhibited good mechanical properties of prepared ODFs. 

**Figure 11:**
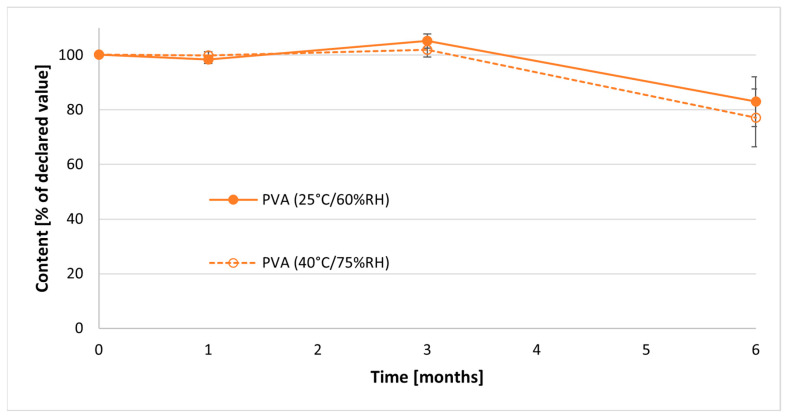
Content of RRE in prepared PVA-based ODFs during stability studies.

Regarding the content of RRE in PVA-based films, there was no significant change of the content during first 3 months of the study. However, similarly as in the case of tablets, a significant decrease in API amount was observed after 6 months of the study (Figure 11). In the films stored at 25 °C/60%RH, the content of the rhinacanthin decreased to approximately 83%, while in films stored at higher temperature and humidity, to 77% of the content compared to films at time zero.

## 3. Discussion

Herbal medicines are the most traditional form of drugs, used for many thousands of years since the beginning of the human history [39]. They were traditionally used as a whole or part of dried plants, tinctures, elixirs, etc. Later, when modern methods of extraction and analytical techniques were developed, single active substances started to be isolated from the plants. They also become intermediate products for many drug substances. Lastly, biotechnological methods were involved in the increasing of the number of active substances obtained from plants or improving a profile of plant extracts. Despite numerous studies on herbal medicines and their long traditional use, there are still numerous gaps and low availability of modern dosage forms containing natural compounds. In the era of growing awareness of sustainability, such drugs become high-demand products, and if patients have the possibility to choose, most of them will select natural products rather than synthetic ones if they only have a similar pharmacodynamic profile. What is more, most of natural herbal products have a wide potential of activity, and therefore they are tested against multiple conditions as main or supplementary compounds [39].

*Rhinacanthus nasutus* has been traditionally used for the treatment of skin diseases, but recently, its antidiabetic activity was discovered and commercialized in the form of infusions or teas [23,25]. Because the number of active substances, namely, rhinacanthins, in such dosage forms may vary significantly and depend on the preparation method, and there is a high demand to develop more patient convenient dosage forms, available for the wide variety of the population, including children and elderly, our aim was thus to design orodispersible dosage forms with rhinacanthin-rich extract (RRE) previously developed by part of our research group. Such dosage forms can help to popularize the usage of rhinacanthins in the therapy of diabetes and treatment of its complications, since rhinacanthins are also considered as having nephroprotective activity due to their antioxidative effect [24].

Major formulary problems encountered during the development of orodispersible dosage forms include taste masking, achieving relatively short disintegration time along with sufficient mechanical resistance, and appropriate stability. Therefore, our research was focused mainly on these properties of ODTs and ODFs. 

In the case of tablets, we encountered problems with poor compressibility of the RRE, which is quite a common problem of plant origin substances. Even if they are of good chemical quality and purity, their physical characteristics may exhibit large batch-to-batch or even within-batch variability. This was also the case in our research. The very first trials on tableting showed us that extract is not uniform. The microscopic analysis revealed a huge difference in particles’ sizes and shapes, which led to non-uniformity in the tablet mass and related problems with their properties. Calibration of the extract particle size by simple sieving improved the properties of tablets, so we finally used only sieved extract. Despite that, its compressibility properties were far from satisfactory. Therefore, formulation of ODTs included preparation of many preliminary series to optimize the composition of the final product. We tested four different co-processed excipients, finally selecting Pharmaburst as the best one for this extract. 

The prepared optimized series of tablets were of relatively low mechanical resistance. However, it was sufficient in terms of compendial requirements, and appropriate friability was achieved, despite tensile strength as low as 0.45 and 0.62 MPa. Nevertheless, one of the most important parameters of ODTs is their disintegration time. In the case of 10 mm tablets, i.e., containing 50 mg of extract, disintegration time was 38.7 s, while in the case of tablets with 100 mg of RRE, it was 54.2 s. Both of them fully complied with European Pharmacopoeia requirements of less than 3 min.

Despite preliminary problems with a non-uniformity of an extract, the uniformity of the final formulations was on a very good level, as proven in the uniformity of mass, content, and single-unit dosage forms test. Application of the proper mixing technique including step-by-step dilution of the excipients during mixing allowed us to deal with this problem. 

In accordance with the European Pharmacopoeia monograph, it is imperative for ODFs to possess adequate mechanical strength to endure handling without succumbing to damage. As highlighted in the literature [40], an ideal ODF should exhibit moderate tensile strength, low Young’s modulus, and a high percentage of elongation. The acceptance criteria, stipulating a Young’s modulus of <550 MPa, tensile strength (TS) > 2 MPa, and elongation at break (E%) > 10%, were strictly adhered to [41]. All prepared films successfully met these predetermined standards; however, discernible variations in their mechanical properties were observed, contingent upon the specific type and quantity of polymers and plasticizers utilized. The outcomes of conducted tests underscored the influence of plasticizer type and concentration on key mechanical parameters, namely, tensile strength (TS), elongation (E), and percentage of elongation (E%). Notably, an augmentation in the quantity of plasticizer corresponded to a proportional increase in the percentage of elongation, tensile strength, and resistance to probe displacement.

The variations in the wettability of the films were noted based on the film-forming polymer and plasticizer. Although the nature of the films was similar, differences in the preparation method were found to impact the wettability of the ODFs. PVA, being a water-soluble synthetic polymer, possesses versatile properties and is advantageous in various medical applications due to its biocompatibility, low propensity for protein adhesion, and minimal toxicity. Conversely, Lycoat, a natural polymer, contributes film-forming and coating properties to various oral dosage forms in both pharmaceutical and nutraceutical contexts, presenting itself as a potential substitute for other synthetic polymers, owing to its favorable safety profile [20].

As per the European Pharmacopoeia monograph on ODFs, they are expected to disintegrate rapidly in the oral cavity. However, the pharmacopoeia does not specify a maximum threshold for disintegration time or prescribe a standardized method for its determination. Consequently, two distinct methodologies, employing a pharmacopoeial apparatus and a slide frame and ball method, were employed to assess the disintegration time of the prepared ODFs. The precise underlying mechanism related to the slide frame and ball method phenomenon remains unclear; nevertheless, it is postulated that an optimal level of sorbitol, achieving saturation of wettability, is reached at 18% in the formulation. Conversely, the pharmacopoeial method depicted a U-shaped trend with increasing sorbitol content.

Taste masking in orodispersible dosage forms may be one of the most challenging issues in their development. It is especially important when the drug is intended to be administered to pediatric patients, which is very often a case in these preparations. The simplest taste-masking approaches include addition of sweeteners, flavorants, or bitter taste inhibitors. However, it is common that they are not sufficient to mask a taste of many active pharmaceutical ingredients. What is more, even if the taste is masked in an acceptable manner, there may appear unpleasant aftertaste sensations, grittiness, and sandiness in the texture of the powder, etc. Evaluation of taste may be another challenge, and even human trials are haunted with huge variability of the results. In our research, we have used an electronic tongue to perform taste evaluation for selected formulations of ODFs and ODTs. These analyses showed the significant taste-masking effect in both formulations. Chemical images of orodispersible formulations with the addition of sweetener and flavorings were much closer to the chemical images of placebos than chemical images of formulations with RRE but without taste-masking excipients. The addition of sucralose as a sweetener and menthol with mint flavor as taste-masking agents was sufficient to mask a RRE taste in the case of tablets. 

Stability studies of ODTs showed high susceptibility of both tablet formulations to harsh storage conditions. Their physicochemical parameters were significantly worsened during the storage and exceeded beyond the acceptable limits. Moreover, the appearance of the tablets stored in the higher temperature and humidity has also changed. Some discoloration appeared on their surface. Changes were detected also in the case of tablets stored in the temperature 25 °C and 60% humidity, but they were less pronounced. This discoloration might be the effect of high humidity, which affected the partial dissolving of the RRE and its slow diffusion in the tablet mass. The RRE has an intensive red color, and its fresh solutions are red. The probability that the humidity was the main factor affecting stability of the extract can be supported by the literature data. Puttarak et al. [42] reported the susceptibility of the rhinacanthins to light and increased temperature in a dry state, which resulted in its stability for no more than 8 weeks. What is more, the solution of the RRE adjusted to a pH from 5.5 to 8.0 was stable for less than 2 weeks, which showed its high instability when combined with water. In our stability studies of ODTs, we used only standard PVC/Alu blisters, which are highly permeable to water vapor. Much better stability was achieved in the case of ODFs that were stored in the Alu/Alu sachets, forming a much better barrier to humidity. Hence, discoloration of ODTs might occur as a result of inadequate packaging. Utilizing Alu/Alu blister packaging as the primary option could potentially enhance the stability of ODTs compared to PVC/Alu packaging. Active pharmaceutical ingredients prone to the photodegradation can be protected in non-modified tablets by the coating of tablets with polymer film with pigments and opacifiers, which significantly reduce the exposure of the substance to light and minimize the risk of photodegradation. However, such an approach is impossible in the case of ODTs because they cannot be coated. Therefore, the common practice is their packaging into the Alu/Alu blisters. The future formulary studies on the rhinacanthin containing dosage forms should include more emphasis on the aspect of risk of photodegradation and oxidation of the RRE and involve stability testing of the formulations in high-barrier materials.

According to the stability study of ODF containing the ternary complex of RRE, unfortunately, films formulated using Lycoat between 1 and 6 months under all conditions were found to be too sticky to be tested for their properties. Therefore, only PVA films were analyzed for their properties during the stability study. Although Lycoat provides physical and mechanical properties for ODF, its moisture uptake capacity has to be considered. A lower amount of plasticizer in formulation might alleviate high moisture during storage.

The stability study of PVA films yielded promising outcomes, establishing PVA as a viable filming agent based on its commendable physical and mechanical properties, along with favorable disintegration times. The ODF formulations demonstrated rapid disintegration under simulated saliva conditions at 37 °C, with mean disintegration times consistently below 200 s. This swift disintegration aims at enhancing the overall comfort and convenience of administration [43].

The Young’s modulus values, indicative of material stiffness, were influenced by factors such as moisture content and plasticizers, which enhance the mobility of polymer chains. Furthermore, an augmentation in the solid content within the ODF resulted in elevated Young’s modulus values. Simplified stability studies over a 6 month duration revealed an increase in Young’s modulus, emphasizing the impact of the stability period on the mechanical characteristics of the ODF [44]. Despite the visible changes in the values of all mechanical parameters, i.e., Young’s modulus, elongation, and tensile strength, during the storage period, films met the acceptance criteria proposed by Visser et al. [41].

Analysis of the content of rhinacanthin in ODFs indicated that its amount remained at a comparably constant level for 3 months of storage. However, after 6 months, the significant reduction of content was noticed. It could have resulted from the fact that rhinacanthins are photosensitive and prone to oxidation. All formulations were prepared under ambient light and packed in air atmosphere, without the removal of oxygen. The mechanism of RRE degradation in the final dosage form, as well as the effect of more barrier containers on the stability, will be the topic of future investigations. 

In conclusion, the comprehensive 6 month stability investigation demonstrated minimal distinctions in the mechanical characteristics of the studied ODFs. While all ODF formulations exhibited homogeneity and favorable mechanical properties for easy handling, challenges pertaining to moisture and active pharmaceutical ingredient (API) stability were identified. The stability assessments, conducted in accordance with International Council for Harmonization (ICH) guidelines under two storage conditions (25 °C/60% and 40 °C/75% relative humidity), aimed to offer nuanced insights into the variations in formulation over the specified period.

## 4. Materials and Methods

### 4.1. Materials

Co-processed excipients Ludiflash (BASF, Ludwigshafen, Germany), Pharmaburst 500 (SPI Pharma, Wilmington, DE, USA), Ultraburst (SPI Pharma, Wilmington, DE, USA), and F-Melt type C (Fuji Chemical Industries Co., Ltd., Toyama, Japan) were kindly gifted by their manufacturers. Crospovidone–Polyplasdone XL-10 (Ashland, Schaffhausen, Switzerland), croscarmellose sodium–Primellose (DFE Pharma, Goch, Germany), and sodium starch glycolate–Primojel (DFE Pharma, Goch, Germany) were used as superdisintegrants. Microcrystalline cellulose–Vivapur PH-101 (JRS Pharma, Rosenberg, Germany) and Vivapur PH-102 (JRS Pharma, Rosenberg, Germany), D-mannitol–Pearlitol 100SD (Roquette, Lestrem, France), and spray-dried lactose–Supertab 14SD (DFE Pharma, Goch, Germany) were used as fillers. Silicon dioxide–Aerosil 200 (Evonik, Essen, Germany), sodium stearyl fumarate–Pruv (JRS Pharma, Rosenberg, Germany), calcium silicate–Rxcipients FM 1000 (Evonik, Havre de Grace, MD, USA), and magnesium aluminometasilicate–Neusilin US2 (Fuji Chemical Industries Co., Ltd., Toyama, Japan) were used to improve lubrication, flow, and compressibility of the tablet mass. Pregelatinized hydroxypropyl pea starch–Lycoat RS 720^®^ (Roquette, Lestrem, France) and poly(vinyl alcohol)–PVA–Poval 4-88 (Kuraray, Tokyo, Japan) were used as film-forming polymers. Sorbitol–Polysor^®^ (Roquette, Lestrem, France) was used as a plasticizer. Sucralose–Emprove (Merck, Darmstadt, Germany) was used as a sweetener. Menthol (Fagron, Krakow, Poland); citric acid (Chempur, Piekary Slaskie, Poland); bitter masking FLV PDR (Kerry Ingredients and Flavo, Mozzo, Italy); and mint, orange, lemon, strawberry, milk, and honey flavors (Firmenich, Genas, France) were used to mask the taste and smell of RRE.

### 4.2. Preparation of Ternary Complex of Rhinacanthin-Rich Extract

Fresh *R. nasutus* roots were obtained hydroponically, following established procedures, and a voucher specimen (No. 0011814) was stored at the herbarium of the Faculty of Pharmaceutical Sciences, Prince of Songkla University, Thailand. Roots were cleaned, dried, ground into powders, and filtered. *R. nasutus* root extract (RRE) was obtained using ethanol, with a microwave-assisted extraction and fractionation using an Amberlite^®^ column. Only ethanol and water were used. Rhinacanthins-C, -D, and -N were isolated through a silica gel column. Validated HPLC assessments were performed according to a previously described method [23,45]. Briefly, the method was performed using a Shimadzu HPLC system (Model LC-20, Shimadzu, Tokyo, Japan) equipped with a LC-20AD pump, a SIL-20A autosampler, and an SPD-M20A photodiode-array detector. The chromatographic system was operated on a 150 mm × 4.6 mm Phenomenex ODS column and eluted with a solution of methanol and 5% aqueous acetic acid (80:20, *v*/*v*) at a flow rate of 1 mL/min. The quantitative UV detection was set at 254 nm and based on calibration curves, showing concentrations of rhinacanthin-C, -D, and -N in RRE (65.78%, 12.62%, and 7.68% *w*/*w*, respectively), which exceeded established levels. Ternary inclusion complexes of RRE/β-cyclodextrin/PVP K30 were produced using solvent evaporation. A 1:1 ratio of β-cyclodextrin was added to the RRE solution, stirred for 24 h to form a binary complex solution. Then, 10% *w*/*w* PVP K30 of the solid binary complex was added, and the mixture was stirred for 2 h and dried at 60 °C under reduced pressure. The dried complex was ground and sieved.

### 4.3. Preparation of Tablets

Tablets were prepared on 3 different stages of the study in different batch sizes and with different equipment. 

#### 4.3.1. Development of Preliminary Formulations of Tablets Containing RRE

An entry study on tableting included a preparation of 32 different formulations with RRE using EK0 single punch tablet press (Korsch, Berlin, Germany). Their diameters were 9 mm, 10 mm, or 12 mm, and they had masses from 250 mg to 450 mg. Detailed composition is presented in the Appendix A (Table A1). The amount of RRE in a single tablet was 50 mg or 100 mg. They contained different excipients and different flavorings. 

Preparation of tablet mass included three steps. Firstly, sucralose and flavorants were mixed manually with RRE in a plastic container. Secondly, they were mixed with co-processed excipients, superdisintegrants, and all other excipients besides lubricant. These first two steps took about 5 min. In the last step, the powder mass was mixed with a lubricant for 1 min, and the tablet mass was sieved through the plastic sieve of 0.6 mm. 

Screening of the properties of these preformulations and their optimization by modification of their composition led to the selection of two different formulations of tablets with 50 mg or 100 mg of RRE for manufacturing at a larger scale for further studies.

#### 4.3.2. Preparation of an Optimized Batches of Tablets

Two optimized formulations were prepared using rotary tablet press PH103 (Korsch, Germany) in batches of 500 tablets (Table 5). They contained either 50 mg or 100 mg of RRE, depending on the tablet size, i.e., 10 mm or 12 mm and tablet mass 250 mg or 400 mg, respectively. Pharmaburst 500 was used as a co-processed excipient for a direct compression in both formulations. Other components are enlisted in Table 5. Mixing order was the same as described above, but instead of manual mixing, the stainless-steel cube mixer (Erweka, Langen, Germany) rotating with 10 rpm speed was used.

#### 4.3.3. Preparation of Tablets for Taste Studies Utilizing Electronic Tongue

The third kind of tablet was prepared only for in vitro taste studies. Composition of tablets was based on the formulations T10_50 mg and T12_100 mg, but with some modifications implemented to modify a taste. Placebo formulations contained the same amounts of ingredients as in Table 5 but were free of RRE. Their theoretical mass was 200 mg for 10 mm tablets and 300 mg for 12 mm tablets.

Another formulation contained RRE but had no addition of sweetener and flavorings (sucralose, mint flavor, and menthol). Their theoretical masses were 237 mg and 380 mg, respectively. All tablets for taste studies were prepared in the same way as preformulations, using single-punch tablet press Korsch EK0.

### 4.4. Preparation of Films

The RRE-loaded ODFs were prepared by a casting method. Lycoat RS 720 or PVA was added to distilled water with sucralose, at room or high (90 °C) temperature, respectively, and the mixture was stirred at 230 rpm using a Heidolph MR HeiTec magnetic stirrer (Schwabach, Germany). Sorbitol at a concentration of 10.0–20.0% was added to the cooled polymer solution and stirred at room temperature for 15 min. Then, menthol dissolved in ethanol, RRE, and mint flavor were added to polymer-sorbitol solution, and the mixture was homogenized for 1 min at 11,000 rpm using a homogenizer. The composition of the film casting solutions is given in Table 6. After removing the air bubbles under the vacuum, the casting solutions were poured on a plastic foil using a motorized film applicator (Elcometer 4340, Manchester, Great Britain), at a thickness of 500 μm, and dried at room temperature for 12 h. Dry films were cut into rectangular strips (1.5 × 1.5 cm or 2 × 3 cm) and a double-bell shape specimen type 5 corresponding to the DIN EN ISO 527-3 standard [46].

To perform the in vitro taste assessment, an additional four film formulations based on the L16 and PVA formulations were prepared. Two of them were free of RRE, while another two contained only film-forming polymer, plasticizer, and API. All films were prepared in the same way as described above.

### 4.5. Mechanical Parameters of Tablets

The assessment of ODT hardness employed a VK200 tablet tester (Vankel, Cary, NC, USA). It was performed according to the European Pharmacopoeia 11.0 monograph 2.9.8. Resistance to crushing of tablets and USP monograph <1217> for 6 tablets of each formulation [32,47]. Tensile strength (T_s_) was calculated according to the following equation from USP:

T_s_ = 2F/πDH

where F is a breaking force measured with a resistance to crushing test, D is a diameter of tablet in millimeters, and H is a tablet thickness expressed in millimeters.

Friability, in accordance with the European Pharmacopoeia 11.0 monograph 2.9.7., was determined by weighing at least 6.5 g of tablets devoid of dust particles [33]. These tablets were then subjected to 100 cycles in a friabilator drum, after which their weight was measured again. The percentage friability was subsequently calculated based on the percentage loss of their initial mass.

### 4.6. Disintegration Time of Tablets

Two distinct methodologies were employed for the assessment of tablet disintegration time. The first involved the implementation of a standard pharmacopoeial test (according to monograph 2.9.1.), utilizing the disintegration tester ED-2 (Electrolab, Mumbai, India) within approximately 800 mL of distilled water, maintained at a temperature of 37 °C. Six tablets were positioned in tubes within the basket of the disintegration apparatus, and initiation of the test involved vigorous shaking of the basket beneath the water surface, facilitating tablet disintegration. The measurement process was semi-automatically terminated, and the disintegration times for each formulation were subsequently calculated and presented as mean values along with their corresponding standard deviations.

A secondary disintegration methodology involved the utilization of an in-house constructed apparatus (BJKSN-13), designed to measure disintegration time and generate disintegration profile curves, delineating changes in tablet thickness over time [35]. The procedural overview encompasses the placement of a tablet onto a perforated stainless-steel plate, moistened with about 0.8 mL of medium (distilled water) maintained at 37 °C, and subsequently subjected to grinding via a rotating shaft. The registration mechanism was based on a magnetic ring and a Hall effect sensor. The experimentation was iterated six times, with subsequent computation of the arithmetic mean and standard deviations.

### 4.7. Film Thickness and Weight

The assessment of film thickness was conducted utilizing a micrometer screw (Digimatic Micrometer, MDC-25SB, Mitutoyo, Kawasaki, Japan) with a precision of 0.001 mm, and subsequent calculation of the average thickness ensued. Additionally, film weight measurements were undertaken by employing an analytical balance (AS 160/X, Radwag, Krakow, Poland) with a precision of 0.1 mg.

### 4.8. Mechanical Parameters of Films

The evaluation of film mechanical properties utilized an EZ-SX texture analyzer (Shimadzu, Kyoto, Japan). Condition parameters were set as follows: a 500 N load cell and an extension speed of 50 mm/min (sample type 5, n = 5). Three parameters, namely, Young’s modulus, tensile strength (TS), and elongation at break (%E), were analyzed using Trapezium X software (Shimadzu, Kyoto, Japan).

### 4.9. Contact Angle

A DSA255 drop shape analyzer (Krüss, Hamburg, Germany) was used to determine the wettability of ODFs using the sessile drop technique. A 2 μL droplet of the distilled water was deposited on the surface of the ODFs. The contact angle was measured at the 1st and 10th points of contact. All the measurements were repeated three times.

### 4.10. Disintegration Time of Films

The disintegration time of the films was assessed using two methods adapted from Speer et al. [48]. In the first approach, film samples (n = 3) measuring 1.5 × 1.5 cm were secured in a holder featuring a 10 mm diameter circular hole. A stainless-steel ball weighing 3.5 g and measuring 10.0 mm in diameter, along with 900 μL of distilled water at 37 °C, were placed on the film surface. The time taken for the ball to penetrate through the film was recorded.

In the second method, film samples sized 2 × 3 cm were loaded with 3.0 g magnetic clips and placed into the holder clips attached to tubes of a pharmacopoeial disintegration apparatus type A (ED-2 SAPO, Electrolab, Mumbai, India). The test was conducted using 900 mL of distilled water at 37 °C, and the disintegration time was recorded when the bottom clips fell away. Both methods were applied to evaluate six samples of ODFs.

### 4.11. Uniformity of Mass and Drug Content

Ten RRE tablets were weighed using analytical balance MS 105DU (Mettler-Toledo, Greifensee, Switzerland). Their average mass and standard deviations were calculated. Ten RRE tablets were dispersed separately in 100 mL of distilled water, using a laboratory shaker Unimax 1010 (Heidolph Instruments GmbH & Co., Schwabach, Germany). After shaking for 1 h with 400 rpm, samples were filtered and analyzed spectrophotometrically at a wavelength of 278 nm with UV 1900 (Shimadzu, Kyoto, Japan). The mean RRE content and standard deviations were calculated based on the calibration curve for the pure RRE.

A similar analysis was performed for ten units of RRE ODFs of 2 × 3 cm. They were dissolved separately in 50 mL of distilled water, filtered, and analyzed spectrophotometrically at a wavelength of 278 nm with UV 1900 (Shimadzu, Kyoto, Japan). The mean RRE content and standard deviation were calculated.

### 4.12. Evaluation of Taste-Masking Effectiveness Using Electronic Tongue

#### 4.12.1. Electronic Tongue–Sensor Array Fabrication 

For taste-masking efficiency evaluation, the laboratory version of the potentiometric electronic tongue developed at Warsaw University of Technology was used. This system had been previously applied in a number of various pharmaceutical applications based on sensing bitter APIs [49]. Sensor array was composed of 8 types of ion-selective electrodes (ISE), equipped with chemosensitive membranes, whose composition is listed in Table 7. Two electrodes were prepared for each membrane type. 

**Figure 12:**
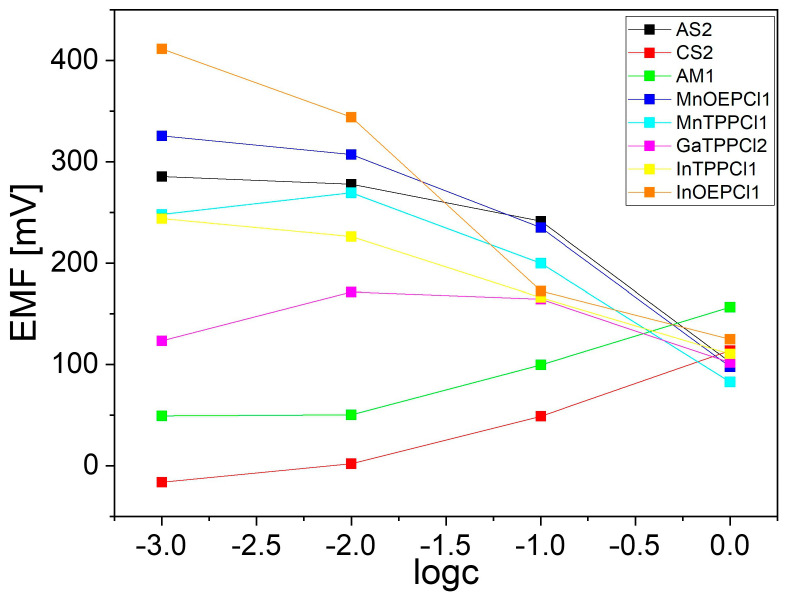
Signals of the electronic tongue sensors depending on RRE concentration.

To ensure appropriate performance of the device, all sensors were tested towards RRE. Stock solutions (1% *w*/*v*) and diluted solutions (0.1–0.001% *w*/*v*) of RRE were used for calibration measurements. The signals of electrodes immersed in solutions of the RRE of increasing concentration were recorded and respective calibration curves are presented in Figure 12.

All electrodes were characterized by high sensitivity towards the tested substance in a varied linear range of the sensor response. Linear regression parameters for the ISEs are presented in Table 8. In some cases, super-Nernstian response was observed, which indicates the complex nature of the membrane response. Nevertheless, high and varied sensitivity of the electrodes towards RRE indicates the possibility of using the fabricated sensor array for the analysis of RRE formulations.

#### 4.12.2. Electronic Tongue–RRE Formulation Measurements

The procedure applied for electronic tongue (e-tongue) analysis is a standard measurement protocol applied previously for various pharmaceutical samples to study taste-masking efficiency [49]. It is based on the following steps: signal stabilization for 5 min (sensors immersed in deionized water); the introduction of the studied pharmaceutical formulation to the medium; and recording of electrodes’ signals that are influenced by the released API and excipients, in time. The signals of the sensors were registered for 10 min (5 min stabilization, 5 min release). Between sample measurements, the ISEs were washed with purified water and dried. The resulting signals were processed using principal component analysis (PCA) performed in SOLO^®^ software, version 8.9 (Eigenvector Research Inc., Wenatchee, WA, USA).

### 4.13. Stability Studies

The simplified stability studies were performed for the ODTs and ODFs placed in two different packages with different permeability for humidity and oxygen. ODTs were packed in standard PVC/aluminum blisters, while ODFs were sealed in aluminum sachets. Both types of formulations were stored under two different conditions for climatic zone II in accordance with the International Council for Harmonization (ICH) guidelines, i.e., 25 °C/60% RH and 40 °C/75% RH for 6 months in the same Memmert type HPP 108 constant climate chambers (Memmert, Schwabach, Germany).

Stability assessment included testing of a disintegration time of ODTs with the pharmacopoeial method and their hardness after 2 weeks and 1, 2, 3, and 6 months; content of RRE after 1, 3, and 6 months; and friability after 6 months. Additionally, disintegration time was measured with the BJKSN-13 apparatus after 1, 3, and 6 months.

For ODF stability assessment, physicochemical properties, mass, thickness, mechanical properties, disintegration time, and content uniformity were determined after 1, 3, and 6 months.

### 4.14. Statistical Analysis of Data

All descriptive statistics were calculated with Microsoft Excel 365 MSO or OriginPro 2020b. In order to identify statistically significant differences, a one-way analysis of variance (ANOVA) with a post hoc Tukey’s multiple comparison test was performed using the Statistics module of the OriginPro 2020b software. Results were considered to be significantly different when *p* < 0.05.

## 5. Conclusions

The results of this study showed that it is possible to formulate orodispersible dosage forms, such as tablets and films, with RRE, having good mechanical properties and short disintegration time. The most promising formulations were PVA-based ODFs. Compared to the other tested orodispersible formulations, i.e., Lycoat-based ODFs and ODTs, they met all the assumed requirements, both immediately after preparation and in the stability tests. They were characterized by low values of Young’s modulus, high percentage of elongation, and moderate tensile strength, regardless of the time and condition of storage. The disintegration time during the stability tests increased slightly; however, it did not exceed the compendial value of 3 min.

Due to the problems with stability of ODTs stored in the standard PVC/Alu blisters, future research will focus on improving their stability by utilizing more barrier packing materials, such as Alu/Alu blisters or protection from the oxidation by modified atmosphere packaging (nitrogen purge packaging). The Alu/Alu blisters are expected to protect the RRE from both photodegradation and contact with humidity as well as reduce the risk of oxidation of the extract. The other aspects of the future formulary studies may include analysis of the compatibility of excipients with the extract and more detailed studies on the mechanisms of its degradation. The next stage of the research may also involve testing of the antidiabetic activity of the tablets and films in animal models.

## Figures and Tables

**Table 1:** Properties of optimized ODTs (average values ± SD).

Parameter	Formulation	
	Tablets 10 mm	Tablets 12 mm
	(50 mg of RRE)	(100 mg of RRE)
Thickness [mm]	3.29 ± 0.17	3.58 ± 0.03
Mass [mg]	248.98 ± 4.98	395.55 ± 7.20
Hardness [N]	32.1 ± 5.5	30.1 ± 3.0
Tensile strength [MPa]	0.62 ± 0.11	0.45 ± 0.04
Friability [%]	0.75	0.53
Disintegration time–FP [s]	38.7 ± 3.1	54.2 ± 3.1
Disintegration time–BJKSN [s]	137.3 ± 11.6	N/A
Content of RRE [mg]	49.85 ± 0.79	99.67 ± 0.78
Content [% of theoretical value]	99.70 ± 1.57	99.67 ± 0.78
Acceptance value (AV) based on the content uniformity	1.88	1.87
Acceptance value (AV) based on the mass uniformity	2.10	4.35

**Table 2:** Properties of ODFs (average values ± SD).

Formulation	Weight [g]	Thickness [mm]	Elongation [%]
L10	0.149 ± 0.01	0.207 ± 0.01	1.97 ± 0.3
L12	0.159 ± 0.01	0.214 ± 0.02	1.81 ± 0.7
L14	0.174 ± 0.01	0.230 ± 0.01	26.80 ± 7.2
L16	0.174 ± 0.01	0.231 ± 0.01	21.05 ± 5.9
L18	0.184 ± 0.01	0.224 ± 0.01	21.16 ± 5.7
L20	0.166 ± 0.02	0.210 ± 0.01	39.15 ± 9.2
PVA	0.148 ± 0.02	0.222 ± 0.01	94.23 ± 20.1

**Table 3:** Wettability of ODFs (average values ± SD).

Formulation	Contact Angle [°]	
	At the 1st Point	At the 10th Point
L10	65.4 ± 3.1	56.4 ± 2.8
L12	70.7 ± 2.6	59.4 ± 1.5
L14	69.3 ± 1.4	57.1 ± 0.6
L16	66.3 ± 1.3	53.1 ± 2.5
L18	69.0 ± 2.5	48.8 ± 1.9
L20	62.0 ± 0.9	49.2 ± 0.5
PVA	57.0 ± 0.9	47.3 ± 0.0

**Table 4:** Properties of PVA-based ODFs in the stability test (average values ± SD).

	T0	1 mo		3 mo		6 mo	
		25 °C/60%	40 °C/75%	25 °C/60%	40 °C/75%	25 °C/60%	40 °C/75%
Weight [g]	0.148 ± 0.0	0.147 ± 0.0	0.145 ± 0.0	0.148 ± 0.0	0.148 ± 0.0	0.149 ± 0.0	0.161 ± 0.0
Thickness [mm]	0.222 ± 0.0	0.224 ± 0.0	0.224 ± 0.0	0.211 ± 0.0	0.217 ± 0.0	0.219 ± 0.0	0.244 ± 0.0
Disintegration time [s]	137 ± 12	138 ± 5	127 ± 6	109 ± 15	127 ± 6	183 ± 58	152 ± 26
Young’s modulus [MPa]	35.5 ± 5.4	17.6 ± 1.0	26.0 ± 2.3	19.8 ± 1.4	26.7 ± 4.0	61.1 ± 15.1	57.1 ± 6.0
Elongation [%]	105 ± 14	65 ± 16	50 ± 5	40 ± 2	35 ± 2	57 ± 5	51 ± 2
Tensile strength [MPa]	5.42 ± 0.20	3.81 ± 0.81	4.38 ± 0.26	3.52 ± 0.05	3.91 ± 0.01	6.50 ± 0.21	5.67 ± 0.03

**Table 5:** Composition of tablets with RRE.

Ingredient	T10_50 mg		T12_100 mg	
	[%]	[mg]	[%]	[mg]
Rhinacanthin-rich extract (RRE)	20.00	50.00	25.00	100.00
Pharmaburst	59.80	149.50	57.00	228.00
RxCIPIENTS FM1000	12.00	30.00	10.00	40.00
Sucralose	2.00	5.00	1.75	7.00
Mint flavor	2.00	5.00	2.00	8.00
Menthol	1.20	3.00	1.25	5.00
Aerosil 200	1.00	2.50	1.00	4.00
Sodium stearyl fumarate	2.00	5.00	2.00	8.00
Mass [mg]	250		400	
Diameter [mm]	10.0		12.0	

**Table 6:** Composition of ODFs.

Formulation	Content of Film-Forming Solution Components [% (*w*/*w*)]								
	RRE	Lycoat	PVA	Sorbitol	Sucralose	Mint Flavor	Menthol	Ethanol	Water
L10	10.0	25.0		10.0	1.0	0.5	1.0	2.0	50.5
L12				12.0					48.5
L14				14.0					46.5
L16				16.0					44.5
L18				18.0					42.5
L20				20.0					40.5
PVA	10.0	-	25.0	16.0	1.0	0.5	1.0	2.0	44.5

**Table 7:** Sensor array of the applied electronic tongue system.

No	ISE ID	Polymer	Plasticizer	Lipophilic Salt	Ionophore
1	AS	PVC, 66 mg	o-NPOE, 132 mg	TDMAC, 2.0 mg	-
2	CS		DOS, 132 mg	KTFPB, 2.0 mg	-
3	AM		o-NPOE, 132 mg	NaTFPB, 1.2 mg	Lasalocid A sodium salt, 2.2 mg
4	MnOEPCl		o-NPOE, 132 mg	KTpClPB, 0.5 mg	MnOEPCl, 2.0 mg
5	MnTPPCl		o-NPOE, 132 mg	KTpClPB, 0.4 mg	MnTPPCl, 2.0 mg
6	GaTPPCl		o-NPOE, 132 mg	KTpClPB, 0.3 mg	GaTPPCl, 2.0 mg
7	InTPPCl		DOP, 132 mg	KTFPB, 0.3 mg	InTPPCl, 2.1 mg
8	InOEPCl		o-NPOE, 132 mg	KTFPB, 0.3 mg	InOEPCl 1.9 mg

MnOEPCl–2,3,7,8,12,13,17,18-Octaethyl-21H,23H-porphine manganese(III) chloride; MnTPPCl–5,10,15,20-tetraphenyl-21H,23H-porphine manganese(III) chloride; GaTPPCl–gallium(III) 5,10,15,20-(tetraphenyl)porphyrin chloride; GaOEPCl–gallium(III) 2,3,7,8,12,13,17,18-octaethylporphyrin chloride; InTPPCl–indium(III) 5,10,15,20-(tetraphenyl)porphyrin chloride; InOEPCl–indium(III) 2,3,7,8,12,13,17,18-octaethylporphyrin chloride; o-NPOE–2-nitrophenyl octyl ether; DOS–bis(2-ethylhexyl) sebacate; DOP—bis(2-ethylhexyl) phthalate; KTFPB—potassium tetrakis[3,5-bis(trifluoromethyl)phenyl]borate; NaTFPB—sodium tetrakis[3,5-bis(trifluoromethyl)phenyl]borate; KTpClPB—potassium tetrakis(4-chlorophenyl)borate.

**Table 8:** Linear regression parameters of the responses of the electronic tongue sensors towards RRE.

ISE ID	AS	CS	AM	MnOEPCl	MnTPPCl	GaTPPCl	InTPPCl	InOEPCl
Slope [mV/dec]	−60.3	43.7	37.1	−76.8	−56.5	−7.1	−46.0	−103.06
Intercept [mV]	148.7	102.7	144.6	118.4	115.2	129.6	117.6	108.65
R^2^	0.88	0.94	0.89	0.91	0.77	0.08	0.96	0.95

## Data Availability

Dataset available on request from the authors.

## Appendix A

**Table A1:** Composition of the preliminary formulation of tablets prepared with a single punch tablet press.

**Ingredient**	**F1**	**F2**	**F3**	**F4**	**F5**	**F6**	**F7**	**F8**	**F9**	**F10**	**F11**	**F12**	**F13**	**F14**	**F15**	**F16**
Rhinacanthin-rich extract (RRE)	100	100	100	100	100	100	100	100	100	100	100	100	100	100	50	100
Ludiflash	279	204	178	183												
Pharmaburst 500					238	213	215	183	230	246	247	247	268		318	
F-Melt type C														268		
Vivapur PH-101 (microcrystalline cellulose)			20	20				20	20	20	20	20				
Vivapur PH-102 (microcrystalline cellulose)																20
SuperTab 14SD (spray-dried lactose)		50	40	40	20	20	20	40	40							
Pearlitol 100SD (D-mannitol)																210
Polyplasdone XL (crospovidone)		20	25	25		25	25	25	25	45						32
Primellose (croscarmellose sodium)											45					
Primojel (sodium starch glycolate)												45				
Emprove (sucralose)	4	5	10	6	6	10	8	6	7	8	7	7	7	7	7	7
Citric acid		2	5													
Menthol				4	4		4	4	5	5	5	5	5	5	5	
Bitter masking FLV PDR					4											
Strawberry flavor	1				6											
Orange flavor		3	6													
Mint flavor				6	6		6	6	7	8	8	8	8	8	8	15
Milk and honey flavor						16										
Lemon flavor							6									
Aerosil 200–silicone dioxide	8	8	8	8	8	8	8	8	8	9	9	9	4	4	4	8
Pruv (sodium stearyl fumarate)	8	8	8	8	8	8	8	8	8	9	9	9	8	8	8	8
Tablet mass	400	400	400	400	400	400	400	400	450	450	450	450	400	400	400	400
																
**Ingredient**	**F17**	**F18**	**F19**	**F20**	**F21**	**F22**	**F23**	**F24**	**F25**	**F26**	**F27**	**F28**	**F29**	**F30**	**F31**	**F32**
Rhinacanthins-rich extract (RRE)	100	100	100	100	100	100	100	100	100	100	100	100	100	100	50	50
Pharmaburst 500	254	260	180	228	248	228	208	208						188	156	149.5
Ultraburst									268	228	208	260	192			
Vivapur PH-102 (microcrystalline cellulose)														40		
Polyplasdone XL (crospovidone)						20	30	30			30		30			
RxCIPIENTS FM1000 (calcium silicate)				40	20	20	30	30		40	30		30	40	30	30
Neusilin US2 (mangesium aluminometasilicate)			80													
Emprove (sucralose)	7	7	7	7	7	7	7	7	7	7	7	7	7	7	5	5
Menthol		5	5	5	5	5	5	5	5	5	5	5	5	5	4	3
Mint flavor	15	8	8	8	8	8	8	8	8	8	8	8	8	8	6	5
Lemon flavor												8	16			
Aerosil 200–silicone dioxide	8	4	4	4	4	4	4	4	4	4	4	4	4	4	3	2.5
Pruv (sodium stearyl fumarate)	16	16	16	8	8	8	8	8	8	8	8	8	8	8	6	5
Tablet mass	400	400	400	400	400	400	400	400	400	400	400	400	400	400	260	250

**Table A2:** Properties of preliminary formulations of tablets with RRE prepared with single punch tablet press.

		**F1**	**F2**	**F3**	**F4**	**F5 ***	**F6 ***	**F7**	**F8 ***	**F9**	**F10 ***	**F11 ***	**F12 ***	**F13**	**F14**	**F15 ***	**F16**
Disintegration time [s]	AVG.	651.2	775.0	557.4	295.4	175.0	134.6	194.6	112.6	197.6	156.0	136.2	98.2	204.6	452.4	71.2	571.2
	SD	116.8	38.4	48.9	27.7	19.3	6.3	8.7	26.4	7.6	21.8	9.0	21.1	11.8	32.7	4.1	29.7
Hardness [N]	AVG.	41.9	72.6	85.0	41.2	59.5	24.9	53.3	22.6	32.4	13.1	20.9	27.5	28.8	129.8	27.8	34.0
	SD	11.1	5.6	16.5	4.9	14.8	1.7	1.9	3.7	2.1	3.6	1.2	0.8	0.5	0.5	4.9	1.9
		**F17 ***	**F18 ***	**F19 ***	**F20 ***	**F21 ***	**F22 ***	**F23 ***	**F24 ***	**F25 ***	**F26 ***	**F27 ***	**F28 ***	**F29 ***	**F30 ***	**F31 ***	**F32 ***
Disintegration time [s]	AVG.	65.4	139.2	83.8	37.0	88.2	88.0	67.6	70.0	72.0	42.3	63.0	148.2	90.6	122.3	44.3	39.6
	SD	28.0	7.1	5.0	6.6	1.6	3.0	1.8	3.0	1.8	2.5	2.6	5.5	4.2	4.6	3.9	3.5
Hardness [N]	AVG.	81.1	44.8	29.4	28.5	22.9	30.1	38.6	34.0	27.8	26.2	26.2	47.4	30.2	52.1	29.6	33.2
	SD	13.4	1.7	1.6	2.1	1.2	1.2	2.4	2.0	2.0	2.4	3.2	1.7	4.2	6.5	3.7	4.1

* formulations which met the Eur. Pharm. criteria regarding disintegration time (<3 min).

**Table A3:** Properties of 10 mm ODTs during the stability studies.

Rameter	T_0_	25 °C/60% RH					40 °C/75% RH				
		0.5 mo	1 mo	2 mo	3 mo	6 mo	0.5 mo	1 mo	2 mo	3 mo	6 mo
Hardness [N]	32.05 ± 5.47	31.39 ± 5.26	31.23 ± 3.59	32.05 ± 3.60	22.07 ± 4.33	21.26 ± 4.75	33.19 ± 2.87	33.19 ± 1.69	32.05 ± 4.75	16.51 ± 2.44	8.18 ± 0.57
Disintegration time–FP [s]	38.7 ± 3.1	41.8 ± 3.7	40.8 ± 3.3	39.0 ± 4.2	46.8 ± 31.0	58.0 ± 15.9	38.7 ± 4.6	42.8 ± 2.1	42.7 ± 5.4	32.3 ± 4.7	26.8 ± 7.1
Disintegration time–BJKSN [s]	137.33 ± 11.59	-	138.67 ± 13.80	148.00 ± 9.54	104.03 ± 32.02	125.83 ± 22.65	-	137.67 ± 7.77	>180	54.87 ± 6.78	33.00 ± 5.21
Content [% of declared value]	99.70 ± 0.79	-	98.67 ± 0.54	-	83.36 ± 3.04	85.65 ± 6.77	-	97.91 ± 1.46	-	88.15 ± 3.90	80.59 ± 5.60

**Table A4:** Properties of 12 mm ODTs during the stability studies.

Parameter	T_0_	25 °C/60% RH					40 °C/75% RH				
		0.5 mo	1 mo	2 mo	3 mo	6 mo	0.5 mo	1 mo	2 mo	3 mo	6 mo
Hardness [N]	30.08 ± 3.02	42.35 ± 4.78	36.79 ± 3.15	35.48 ± 1.90	23.54 ± 4.64	22.56 ± 1.07	37.61 ± 2.82	33.84 ± 3.15	31.23 ± 3.74	48.07 ± 6.99	26.16 ± 1.48
Disintegration time–FP [s]	54.2 ± 3.1	57.0 ± 5.8	54.7 ± 3.1	58.0 ± 5.8	116.5 ± 29.6	85.2 ± 19.3	57.0 ± 7.6	57.8 ± 4.5	62.5 ± 4.5	93.2 ± 27.6	39.3 ± 8.8
Disintegration time–BJKSN [s]	>180	-	>180	>180	>180	>180	-	>180	>180	150.00 ± 4.73	89.03 ± 11.46
Content [% of declared value]	99.67 ± 0.78	-	98.36 ± 1.96	-	89.66 ± 10.40	84.79 ± 2.71	-	95.34 ± 2.66	-	81.55 ± 5.24	77.31 ± 1.91
